# Rectal Cancer Treatment Management: Deep-Learning Neural Network Based on Photoacoustic Microscopy Image Outperforms Histogram-Feature-Based Classification

**DOI:** 10.3389/fonc.2021.715332

**Published:** 2021-09-23

**Authors:** Xiandong Leng, Eghbal Amidi, Sitai Kou, Hassam Cheema, Ebunoluwa Otegbeye, William Jr Chapman, Matthew Mutch, Quing Zhu

**Affiliations:** ^1^ Department of Biomedical Engineering, Washington University in St. Louis, St. Louis, MO, United States; ^2^ Department of Pathology, Washington University in St. Louis, St. Louis, MO, United States; ^3^ Division of Colorectal Surgery, Department of Surgery, Washington University School of Medicine, St. Louis, MO, United States; ^4^ Department of Radiology, Washington University School of Medicine, St. Louis, MO, United States

**Keywords:** rectal cancer, ultrasound imaging, photoacoustic imaging of rectal cancer, machine learning, regression analysis

## Abstract

We have developed a novel photoacoustic microscopy/ultrasound (PAM/US) endoscope to image post-treatment rectal cancer for surgical management of residual tumor after radiation and chemotherapy. Paired with a deep-learning convolutional neural network (CNN), the PAM images accurately differentiated pathological complete responders (pCR) from incomplete responders. However, the role of CNNs compared with traditional histogram-feature based classifiers needs further exploration. In this work, we compare the performance of the CNN models to generalized linear models (GLM) across 24 *ex vivo* specimens and 10 *in vivo* patient examinations. First order statistical features were extracted from histograms of PAM and US images to train, validate and test GLM models, while PAM and US images were directly used to train, validate, and test CNN models. The PAM-CNN model performed superiorly with an AUC of 0.96 (95% CI: 0.95-0.98) compared to the best PAM-GLM model using kurtosis with an AUC of 0.82 (95% CI: 0.82-0.83). We also found that both CNN and GLMs derived from photoacoustic data outperformed those utilizing ultrasound alone. We conclude that deep-learning neural networks paired with photoacoustic images is the optimal analysis framework for determining presence of residual cancer in the treated human rectum.

## Introduction

Colorectal cancer is the third most common cancer diagnosed in both men and women in the United States (1). While treatment often involves radiation, chemotherapy, and surgical resection, recent advances in neoadjuvant (preoperative) treatment of locally advanced rectal cancers (LARC) have enabled 20-30% of patients to safely avoid surgery altogether (2–5). However, this “watch and wait” approach depends on accurate assessments of tumor regression and high-resolution and high-sensitivity surveillance imaging for tumor recurrence.

Standard surveillance modalities include physical exam, endoscopy with biopsy, and MRI; however, each of these modalities have distinct weaknesses in the post-treatment setting (6–12). Current technology is not able to definitively identify pathological complete responders (pCRs), who may benefit from “watch and wait”, from those with residual disease, who need surgical resection (non-responders).

To overcome these challenges, we developed a co-registered endorectal photoacoustic microscopy and ultrasound (PAM/US) system to assess rectal cancer treatment response (13, 14). Photoacoustic imaging (PAI) is a hybrid imaging technology that uses a short laser pulses to excite hemoglobin molecules endogenous to the human body. The resulting acoustic waves are then acquired by US transducers and analyzed for vascular bed quantification. This process has been utilized in many different areas such as breast cancer (15, 16), lung cancer (17, 18), ovarian cancer (19), skin cancer (20), and colorectal cancer (13, 14). In two recent studies, Cong and colleagues and Liu and colleagues have developed co-registered photoacoustic and ultrasound imaging systems for potential transrectal evaluation of prostate (21, 22).

A convolutional neural network (CNN) is an artificial intelligence algorithm with remarkable capabilities for automated image analysis (23). To quantitatively interpret the large volumes of data acquired by the PAM/US system, we designed and incorporated deep-learning CNN models in the PAM system (PAM-CNN) (14). While our deep-learning PAM-CNN model can accurately assess rectal cancer treatment response, it requires a large training and validation data set. The key question remains if the PAM-CNN outperforms traditional histogram-feature based models. In this study, using 24 *ex vivo* and 10 *in vivo* data sets, we compare the performances of the PAM-CNN and the traditional histogram-parameter-based classifiers in rectal cancer treatment evaluation. Unlike CNN models, a generalized logistic regression (GLM) classifier does not require a large dataset for training and validation, however, imaging features must be extracted and evaluated on their diagnostic accuracy. We have computed five PAM image histogram features and used them to train, validate and test GLM classifiers. The performance of deep learning based CNN models is compared with GLM classifiers. To the best of our knowledge, this study is the first to establish the role of our new deep-learning PAM-CNN approach in rectal cancer evaluation.

## Materials and Methods

### Patients, Specimens, and PAM Imaging

Briefly, 10 participants (mean age, 58 years; range 42 – 68 years; 2 women and 8 men) completed radiation and chemotherapy from September 2019 to September 2020 and were imaged with the PAM/US system prior to surgery. In the *in vivo* study, patients who had previously undergone preoperative treatment with radiation and chemotherapy were imaged *in vivo* before resection.

Colorectal specimens from another group of 24 patients who had undergone surgery were studied *ex vivo* (**Table 1**). All studies were approved by the institutional review board of the Washington University School of Medicine, and all patients provided written informed consent. In the *ex vivo* study, each specimen was evaluated within one hour of surgical resection and prior to formalin fixation.

**Table 1:** Lesion characteristics (24 *ex vivo* colorectal specimens and 10 patients).

Lesion characteristics	
*Ex vivo* colorectal cancer (63 years)*	Adenocarcinoma,T1-T3, n=15
*Ex vivo* treated rectal cancer (63 years)	Residual adenocarcinoma, n=3
*Ex vivo* treated rectal cancer (52 years)	pCR, n=3
*Ex vivo* normal colorectal tissue	18 normal areas from cancer patients and five patients with only normal colorectal tissue available
*In vivo* treated rectal cancer (61 years)	Recurrence tumor, residual tumor, n=6
*In vivo* treated rectal cancer (53 years)	pCR, n=1
*In vivo* normal colorectal tissue	10 normal areas from 7 cancer patients and three patients with only normal rectal tissue available

*Average age of each group.

### PAM/US Endoscope

**Figure 1:**
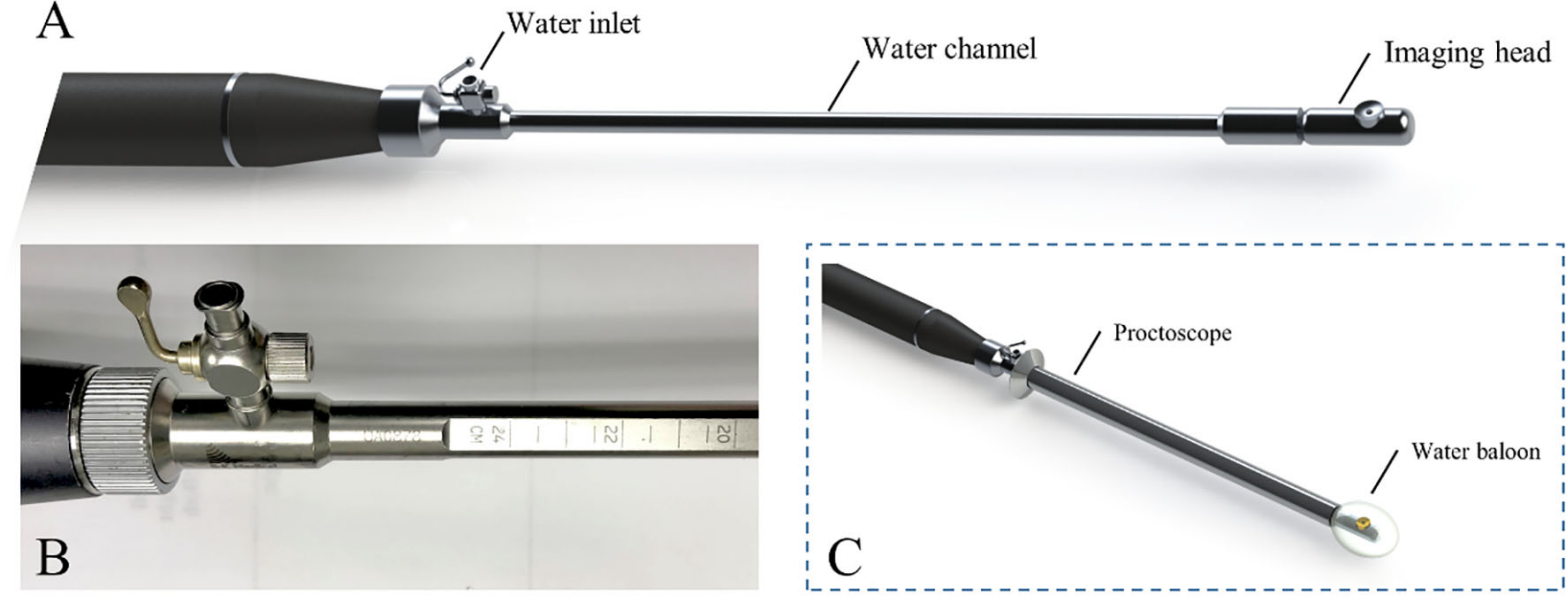
PAM endoscope **(A)**, scales on water channel **(B)** and endoscope in a proctoscope, with a balloon on the tip **(C)**.

The PAM endoscope consists three parts: a handle, a water channel (the main body), and an imaging head, as shown in **Figure 1A** (14). Briefly, the water inlet which allows water injected from a syringe to inflate a water balloon covering the image head for ultrasound coupling. A stepper motor in the handle turns a hollow shaft in the water channel to rotate the image head 360° for full circle imaging. An optical fiber inside the hollow shaft delivers laser pulses to the imaging head. An ultrasonic transducer (20 MHz, 75% bandwidth) fixed on the imaging head both transmits and receives ultrasound signals, and also receives PA signals. An Nd: YAG laser working at 1064 nm with a 1000 Hz pulse repetition rate is the light source. A 0.15 cm^2^ tissue area is illuminated by 3.6 mJ laser pulses from the probe tip, resulting in a surface optical fluence of 24 mJ/cm^2^, which is well within the ANSI safety threshold (100 mJ/cm^2^) at 1064 nm (24). This fluence is further reduced by energy diffusion caused by the balloon.

During imaging, the PAM endoscope is inserted transanally through a proctoscope, (**Figure 1C**). Ruled scales on the water channel (**Figure 1B**) show how deeply the endoscope is inserted into the rectum where the images are obtained. This endoscopy is the first device incorporating PAM into the rectum examination.

### PAM and US Data Selection for Training/Validation and Testing of Models

**Figure 2:**
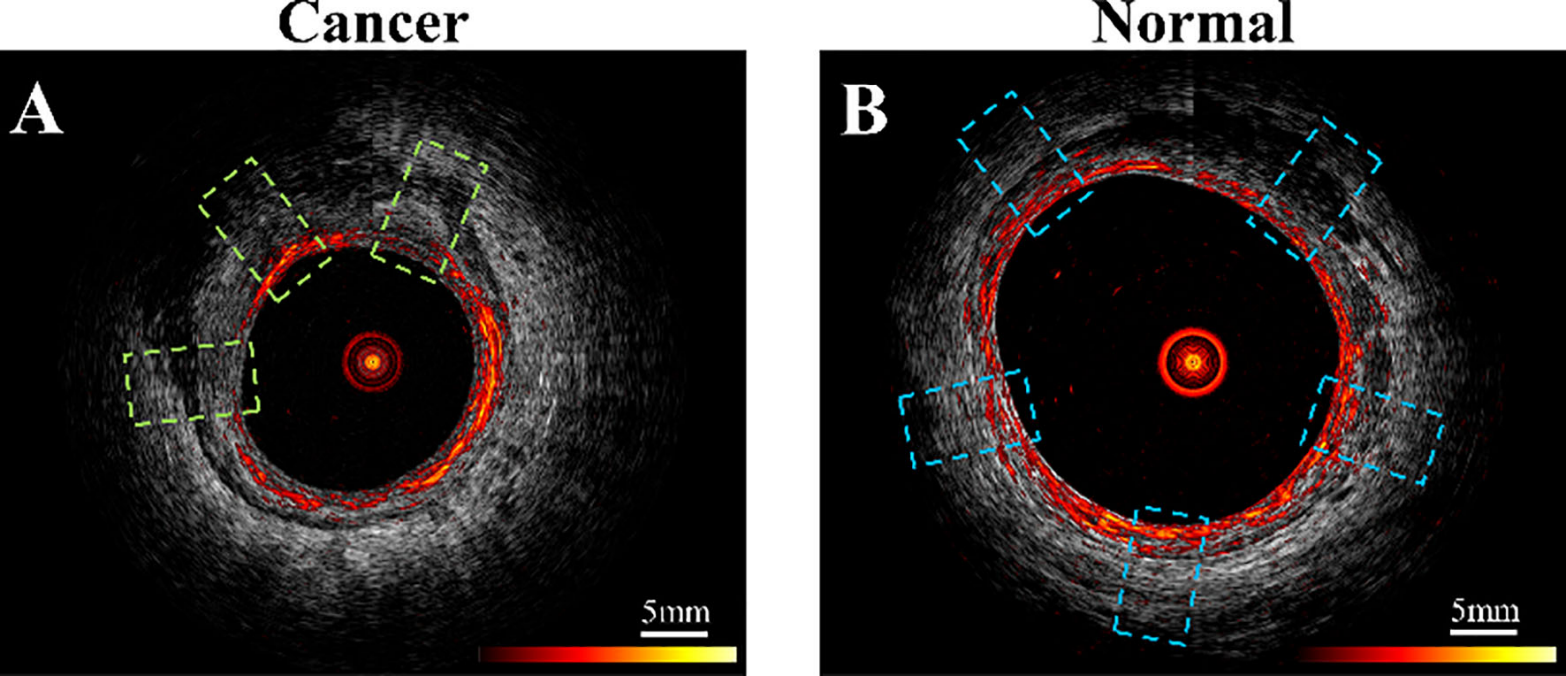
Example co-registered PAM and US images showing ROIs of **(A)** residual cancer tissue, area in green dashed line boxes, and **(B)** normal tissue, area in blue boxes. PAM ROIs are cropped from PAM images, and US ROIs are cropped from US images.

For training and validation, three to five regions of interest (ROIs) were selected at uniformly spaced locations on each PAM or US B-scan image acquired from normal regions or a tumor bed (**Figure 2**). For example, the red ring in **Figure 2** represents mucosa vasculature, which is continuous in the normal image of **Figure 2B**. The blue rectangles indicating ROIs are uniformly spaced along the perimeter of the image. In the cancer image, the dark zones and discontinuities in the red ring from approximately 9:00 to 1:00 o’clock indicate tumor, so the ROIs are uniformly spaced in that segment. A total of 2600 US ROIs (1262 normal and 1496 cancerous) and 2004 PA ROIs (1207 normal and 797 cancerous) were compiled from 24 patients’ *ex vivo* images and 10 patients’ *in vivo* images (**Table 1**). Two *ex vivo* samples showed a low signal-to-noise ratio (SNR) on PAM images due to a laser energy problem. We excluded those two samples in training PAM-CNN and PAM-GLM models. For the US-CNN and US-GLM models, we used all 24 *ex vivo* and all 10 *in vivo* patient data.

**Figure 3:**
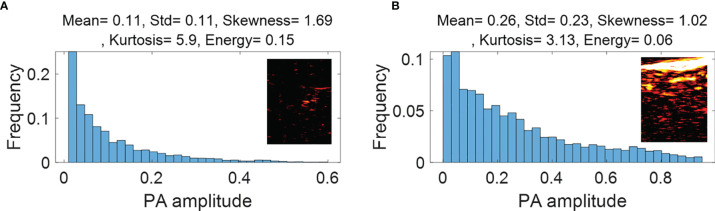
First order statistical features calculated from malignant rectal tissue PAM ROIs **(A)** and normal rectal tissue PAM ROIs **(B)**.

We divided the total of 2004 PA ROIs and total of 2600 US ROIs into two discrete data sets – one for model training and validation and another for testing, respectively. The training set included all *ex vivo* cases (see **Table 1**) and half of the *in vivo* patient data. Of the training set ROIs, 80% were used for training with the remainder for internal validation. The testing set contained the other half of the *in vivo* patient data.

### GLM Models

We used selected image features of ROIs to develop PAM-GLM and US-GLM models. To calculate the histogram of each ROI, we divided the ROI into 32 bins. The bar height of each bin was then computed by dividing the number of pixels with a given value in an associated range by the size of the image. From the histogram of each ROI, we then extracted five features: mean, standard deviation, skewness, kurtosis, and energy.

All the PAM and US features showed significant differences between malignant and normal colorectal tissues (p<0.05) (**Appendix** **Figures 1S** and **2S**). Therefore, all these features were considered as potential candidates when building PAM-GLM and US-GLM models. To prevent model overfitting, the Spearman’s correlation coefficient between each of the histogram features were calculated (**Appendix**, **Table 1S**). We developed PAM-GLM classifiers using each histogram feature separately, as well as using combinations of features with low correlation values. The mean AUCs of the training/validation and testing data sets as well as their 95% confidence of interval were computed for each classifier. The same process was followed to construct US-GLM classifiers.

To remove bias in selecting *in vivo* data for training and validation, we trained the classifiers 10 times. The training/validation and testing data sets are the same as those used for CNN models described in next section.

**Figure 4:**
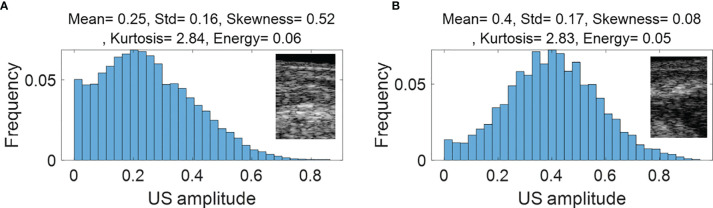
First order statistical features calculated from malignant rectal tissue US ROIs **(A)** and normal rectal tissue US ROIS **(B)**.

**Figure 3** (PAM-GLM) and **Figure 4** (US-GLM) show examples of the first order statistical features calculated from malignant rectal tissue ROIs (shown in **Figure 2A**) and normal rectal tissue ROIs (shown in **Figure 2B**). As shown in **Figure 3**, in PAM ROIs, the malignant tissue has a lower mean and standard deviation, while the other three features are higher. In **Figure 4**, malignant US ROIs show a lower mean and standard deviation than that of the normal US ROIs.

**Figure 5:**
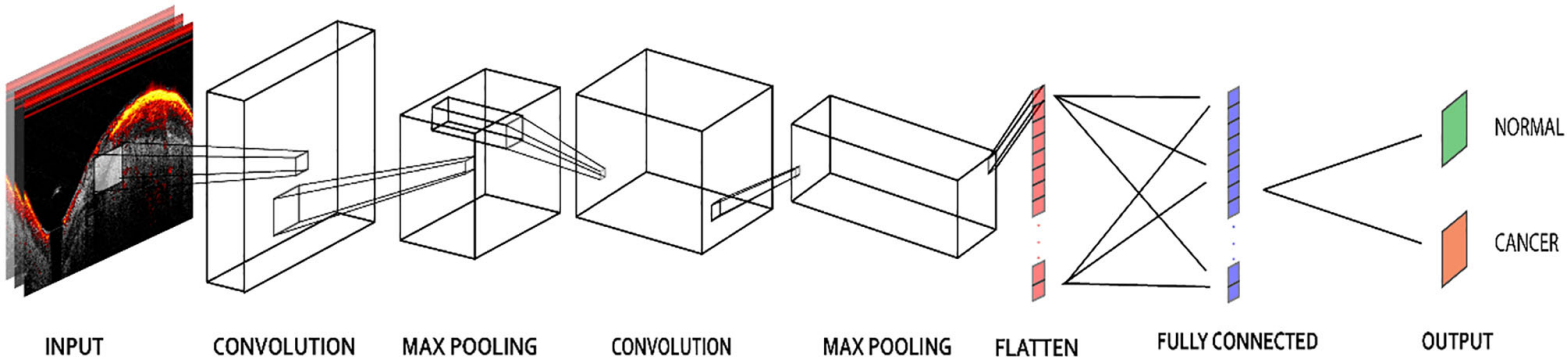
Architecture of the CNN model.

**Figure 1S** in **Appendix** show the boxplots of the histogram features of the PAM ROIs. The p-value for each feature, calculated from a two-sided statistical t-test, is indicated on each plot. All features are statistically significant (p<0.05), however, they are not equally important. To assess the importance of each feature, we first fit a regression model to each feature separately, using all the available data (*ex vivo* and *in vivo* patients), and then we found the AUC of the fitted model. As shown in **Table 2**, Std, Mean, and Kurtosis respectively provide the highest AUC values among all the features of PAM images. While Std and Mean are highly correlated, the correlation value between Mean and Kurtosis is less than 0.5 (**Table S1**). Therefore, these two features are used together to develop PAM-GLM classifiers.

**Table 2:** AUCs of the fitted regression model developed using features of PAM and US images.

Feature	AUC (PAM)	AUC (US)
Mean	0.76	0.81
Std	0.79	0.86
Skewness	0.71	0.57
Kurtosis	0.73	0.62
Energy	0.70	0.85

Similarly, boxplots of the five features from US ROIs are given **Figure 2S**, and the AUC feature values of the fitted model are shown in **Table 2**. Based on this table, Std, Energy, and Mean are respectively the most important features of the US images. However, they all are highly correlated with each other (**Table S2**).

### CNN Models

The PAM-CNN (or US-CNN) architecture (**Figure 5**) contained two sequential feature extraction layers and two fully connected layers (14). Briefly, each extraction layer had a convolutional layer followed by a pooling layer. Each convolutional layer uses a 3 × 3 kernel, and each pooling layer has a 4 × 4 kernel with max-pooling (25). These kernel sizes were selected based on the optimal validation results. The first fully connected layer was a 512-node hidden layer, and the second fully connected layer (output layer) generated two output classifications – normal or cancerous. “Normal” described a layer-like vascular distribution in a PAM image or a layer structure in a US image, and “cancerous” described an absence of the normal vasculature pattern in PAM images, or an absence of the layer structure in US images. A “softmax” activation function in the output layer generated the probabilities of each of the two possible classifications (cancer or normal) for an input image; for each input ROI of a PAM or US image, the CNN model outputted the probability of a normal classification compared to the threshold (e.g. >50% is normal). In all the other layers, a “ReLU” activation function immediately sets all negative values to zero. The ReLU activation function with a gradient of 1 for positive inputs and 0 for negative inputs ensures no exploding or vanishing gradient problem occurs (26).

To avoid biased selection, we trained and validated 10 PAM-CNN and US-CNN models each using all the *ex vivo* data and a randomly selected half of the *in vivo* patient data, while reserving the other half for testing. The maximum number of epochs was 20, with early stopping (a tolerance of 2 epochs) monitored by validation accuracy. If there was no increase in validation for two successive epochs, training was stopped. Stochastic gradient descent was used with a batch size of 20, and the RMSprop optimizer function was used to optimize the neural net weights. The learning rate was set to 10^-3^ with a decay of 10^-5^. In each model, 80% of the ROIs from the training & validation set were used to train the model, the remaining 20% were used for validation, and 20**×** cross validation was performed.

The ROIs of each *in vivo* normal or tumor bed patient images were either all used in training or all used in testing. Each of the 10 CNN models was tested on a randomly selected half of the *in vivo* data and generated an ROC. The overall performance of the classifier was measured by the mean AUC of the 10 models.

The method calculating PAM-CNN’s AUC is different from that of our previous report (14), which leads to a slightly different AUC value. In previous work, the training and validation data set was fixed, which was consisted of 22 *ex vivo* and five *in vivo* data set. The PAM-CNN’s AUC obtained from another five *in vivo* data set unseen by PAM-CNN for testing is 0.98. In this study, we have done a more thorough investigation. The *ex vivo* data set is still fixed for training and validation, but the five *in vivo* data set for training and validation and the five *in vivo* data set for testing were interchanged randomly for 10 times, and the 10 AUC was used to generate the mean value of AUC.

## Results

### GML Models

**Table 3** shows the mean AUCs and 95% confident of interval for PAM-GLM classifiers developed using single features, as well as feature pairs that are weakly correlated (based on **Table S1**). As can be seen, the “Mean-Kurtosis” combination results in a better testing performance than “Mean” alone, and a better training performance than “Kurtosis” alone. In the case of US-GLM (**Table 4**), the classifier which is built using “Std” alone performs best on both training and testing data sets (mean AUCs of 0.86 and 0.66 for training and testing data sets, respectively).

**Table 3:** Training and testing mean AUC values for PAM-GLM classifiers developed using different combinations of weakly correlated features.

Feature combinations	Training AUC (95% CI)	Testing AUC (95% CI)
Mean	0.77 (0.767-0.777)	0.80 (0.793-0.807)
Std	0.79 (0.788-0.793)	0.76 (0.746-0.770)
Skewness	0.71 (0.708-0.719)	0.82 (0.815-0.825)
Kurtosis	0.73 (0.724-0.734)	0.82 (0.817-0.827)
Energy	0.72 (0.712-0.727)	0.74 (0.724-0.758)
Mean, Kurtosis	0.74 (0.732-0.743)	0.82 (0.808-0.820)
Std, Energy	0.80 (0.799-0.807)	0.76 (0.750-0.773)
Kurtosis, Energy	0.75 (0.744-0.750)	0.81 (0.805-0.817)

The 95% confidence of interval values are also shown in front of each mean AUC value.

**Table 4:** Training and testing AUC values for US-GLM classifiers developed using different combinations of weakly correlated features.

Feature combinations	Training AUC (95% CI)	Testing AUC (95% CI)
Mean	0.82 (0.818-0.820)	0.64 (0.629-0.657)
std	0.86 (0.860-0.862)	0.66 (0.650-0.674)
skewness	0.59 (0.587-0.591)	0.42 (0.405-0.443)
Kurtosis	0.64 (0.635-0.639)	0.34 (0.326-0.344)
energy	0.85 (0.851-0.854)	0.61 (0.600-0.621)
Mean, kurtosis	0.82 (0.819-0.822)	0.60 (0.581-0.618)
Std, skew	0.86 (0.860-0.862)	0.65 (0.643-0.664)
Std, kurtosis	0.86 (0.858-0.860)	0.65 (0.642-0.666)
Kurtosis, energy	0.86 (0.856-0.858)	0.63 (0.617-0.638)

The 95% confidence of interval values are also shown in front of each mean AUC value.

**Figure 6:**
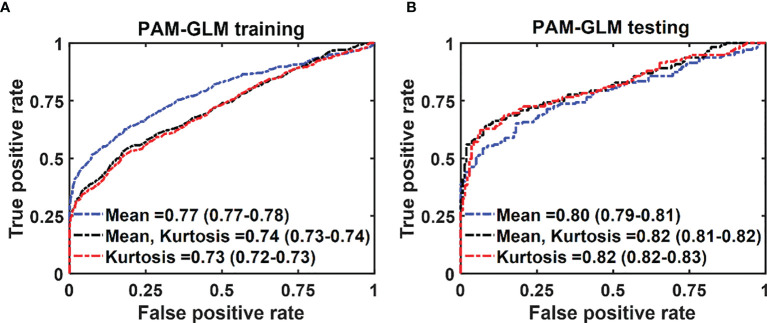
The average ROC of the training **(A)** and testing **(B)** data sets for different combinations of features set. The features were extracted from PAM images. The 95% CIs are indicated in parentheses.

**Figures 6A**, **B** respectively show the mean training and testing ROCs of three of the best performing (based on both training and testing AUCs) classifiers developed using PAM histogram features. As shown in these plots, “Kurtosis” alone results in a slightly better performance on the testing data set than the other feature combinations (see the 95% CI values in the table). It is worth noting that although adding “Mean” to the features set negligibly lowers the AUC of the testing data set, it increases the AUC of the training data set by 0.01. Finally, the reason for the slightly poor training performance than testing for different combinations of features is that the training data set includes both *in vivo* and *ex vivo* ROIs while the testing data set contains only *in vivo* ROIs. Overall, our *in vivo* data have demonstrated slightly better classification between malignant and normal colorectal tissue than the *ex vivo* data.

**Figure 7:**
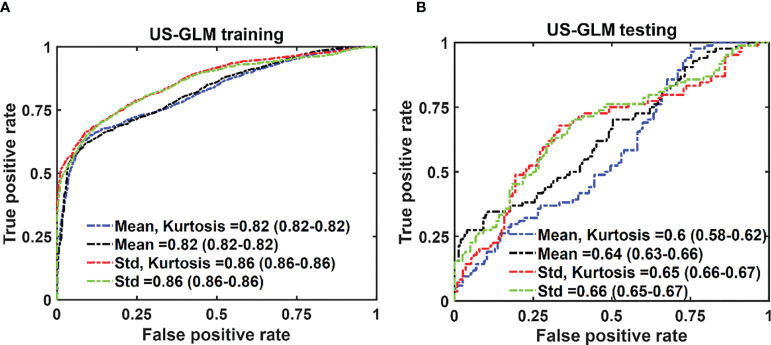
The average ROC of the training **(A)** and testing **(B)** data sets for different combinations of features set. The features were extracted from US images. The 95% CIs are indicated in parentheses.

In the case of US-GLM, using the “Std” histogram feature demonstrates the best prediction AUC of 0.68, as seen in **Figure 7B**. Adding any other uncorrelated features does not improve the AUCs of the training or testing data sets as shown in **Figure 7A**, **B**).

### CNN Models

**Figure 8:**
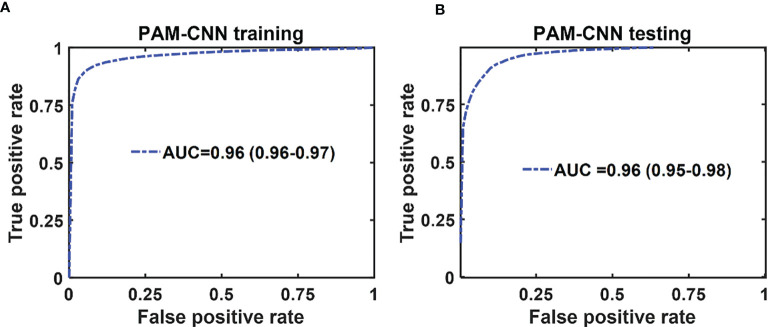
Average ROCs of PAM-CNN model. **(A)** training and validation results, **(B)** testing results. The 95% CIs are indicated in parentheses.

**Figure 9:**
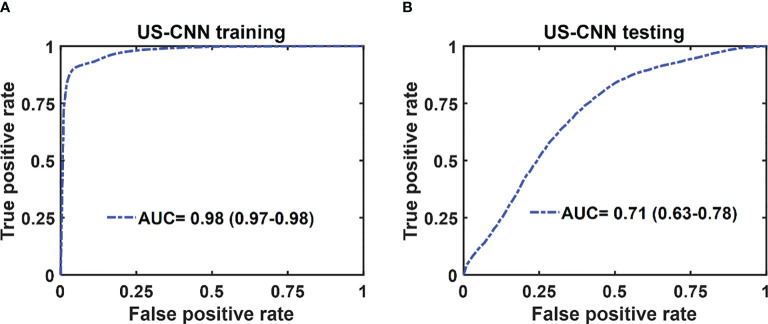
Average ROCs of US-CNN model. **(A)** training and validation, **(B)** testing results. The 95% CIs are indicated in parentheses.

The mean ROC and AUC of the CNN models were computed from 10 CNN models, using the same shuffle method as in GLM. PAM-CNN demonstrated high performance in training and testing, with a 0.96 AUC for both (**Figure 8**). For US-CNN (**Figure 9**), the average AUC was 0.71 in testing.

## Discussion

The general architecture of the normal colon and rectal tissue consists of the mucosa (a thin layer of epithelial cells, a layer of connective tissue, a thin layer of muscle), submucosa (mucous glands, blood vessels, lymph vessels), muscularis propria (a thick layer of muscle), and serosa (an outer layer of the colon). In malignancy, the individual cell types are similar, but the architecture is distorted because cancerous cells of mucosal origin penetrate into the deeper layers of the organ. As these cells invade, the organized structure and vascular network are lost. We have observed uniform, layer-like vasculature with intense photoacoustic signals within normal rectal submucosa and in the tumor beds where complete tumor destruction has occurred. In contrast, heterogeneous and often microvascular-deficient regions have been found consistently in tumor beds with residual cancer at treatment completion (13, 14). The return of a “normal” vascular pattern to the tumor bed appears to signal complete tumor destruction, though this mechanism is not well-understood. As demonstrated, PAM-CNN captures this unique pattern and predicts pCR with a high diagnostic accuracy. PAM-GLM uses first order statistical features extracted from PAM histograms and these features do not contain spatial micro-features that can be learned by deep-learning neural networks. Thus, the performance of PAM-GLM is significantly poorer than PAM-CNNs.

In summary, we have shown in this manuscript that the performance of deep-learning based PAM-CNN models was significantly better than that of the PAM-GLM classifier with AUC of 0.96 (95% CI: 0.95 - 0.98) *vs*. 0.82 (95% CI: 0.81-0.83) using PAM Kurtosis. Both ultrasound-derived models (US-CNN and US-GLM) performed poorly with AUCs of 0.71 (95% CI: 0.63 – 0.78) and 0.66 (95% CI: 0.65 – 0.67), respectively. While easier to train and validate and requiring smaller data sets, GLM diagnostic performance is inferior to CNN models. While the photoacoustic endoscopy system and deep-learning based PAM-CNN models have reported in reference 14, this manuscript is the first to establish the superior role of deep-learning PAM-CNN models in rectal cancer treatment assessment.

Our study has a significant impact in rectal cancer treatment management. The PAM/US endoscopy paired with CNNs has a great potential to improve curent standard of care imaging in accurately predicting complete pathological response (pCR) of rectal cancer post-treatment. For those who have achieved a pCR, unnecessary surgery can be avoided without compromising cancer-related outcomes, and thereby lowering morbidity and health care cost.

Our study has limitations. First, the patient data is limited. With more patient data available, the diagnostic performance of PAM-CNN models can be further improved. For example, in our current study, 1-D ROIs from PAM and US B-scans were used as input images to CNNs. Misclassifications can occur in ROIs’ when SNRs are low. 2-D ROIs from a small number of sequential B-scans can be trained together to reduce the dependence of CNNs on the SNR of individual 1-D ROIs and further improve the performance of CNNs. Second, the quality of *ex vivo* data was not as good as *in vivo* data which can be seen from slightly lower training/validation PAM-GLM data compared with testing results of PAM-GLM. Future studies will be focused on recruiting more patients to the study to further validate the initial results reported in this manuscript.

## Data Availability Statement

The original contributions presented in the study are included in the article/**Supplementary Material**. Further inquiries can be directed to the corresponding author.

## Ethics Statement

The studies involving human participants were reviewed and approved by Institutional Review Board of Washington University School of Medicine. The patients/participants provided their written informed consent to participate in this study. Written informed consent was obtained from the individual(s) for the publication of any potentially identifiable images or data included in this article.

## Author Contributions

Study Design: XL, EA, and QZ. Data acquisition and experiments: all authors Manuscript writing and editing: XL, EA, HC, EO, WC, and QZ. All authors contributed to the article and approved the submitted version.

## Funding

Research reported in this publication was partially supported bythe Siteman Cancer Center and the Foundation for Barnes-Jewish Hospital and by NIH R01 CA237664 and NCI T32CA009621).

## Conflict of Interest

The authors declare that the research was conducted in the absence of any commercial or financial relationships that could be construed as a potential conflict of interest.

## Publisher’s Note

All claims expressed in this article are solely those of the authors and do not necessarily represent those of their affiliated organizations, or those of the publisher, the editors and the reviewers. Any product that may be evaluated in this article, or claim that may be made by its manufacturer, is not guaranteed or endorsed by the publisher.

## Supplementary Material

Supplementary Table 1 Spearman’s correlation between histogram features of the PAM images.

Supplementary Table 2 Spearman’s correlation between histogram features of the US images.

Supplementary Figure 1 Boxplots of histogram features (Y axes) of PAM images. Each plotted point represents the histogram feature in one ROI. The p-value for each feature is shown on the plot.

Supplementary Figure 2 Boxplots of histogram features (Y axes) of US images. Each plotted point represents the histogram feature in one ROI. The p-value for each feature is shown on the plot.

## Appendix

Using highly correlated features to develop a classifier increases the chance of overfitting. To prevent this issue, we calculated the Spearman’s correlation between each two histogram features. **Tables S1** and **S2** show the Spearman’s correlation between each pair of histogram features of the PAM and US images, respectively. These tables are used to select an optimized feature set before developing GLM classifiers.
