# Supplementary material for: Rectal Cancer Treatment Management: Deep-Learning Neural Network Based on Photoacoustic Microscopy Image Outperforms Histogram-Feature-Based Classification
Source: Front Oncol. 2021 Sep 23;11:715332. doi: 10.3389/fonc.2021.715332 (PMC8495416; doi:10.3389/fonc.2021.715332)
Supplement: Supplementary Table 1 — Spearman’s correlation between histogram features of the PAM images. [file Table_1.docx]

Table S1: Spearman’s correlation between histogram features of the PAM images

|  | **Mean** | **Std** | **Skewness** | **Kurtosis** | **Energy** |
| --- | --- | --- | --- | --- | --- |
| **Mean** | 1 | 0.79 | 0.72 | 0.42 | 0.79 |
| **Std** | - | 1 | 0.75 | 0.57 | 0.52 |
| **Skewness** | - | - | 1 | 0.9 | 0.77 |
| **Kurtosis** | - | - | - | 1 | 0.5 |
| **Energy** | - | - | - | - | 1 |
