# Supplementary material for: Rectal Cancer Treatment Management: Deep-Learning Neural Network Based on Photoacoustic Microscopy Image Outperforms Histogram-Feature-Based Classification
Source: Front Oncol. 2021 Sep 23;11:715332. doi: 10.3389/fonc.2021.715332 (PMC8495416; doi:10.3389/fonc.2021.715332)
Supplement: Supplementary Figure 1 — Boxplots of histogram features (Y axes) of PAM images. Each plotted point represents the histogram feature in one ROI. The p-value for each feature is shown on the plot. [file Image_1.pdf]

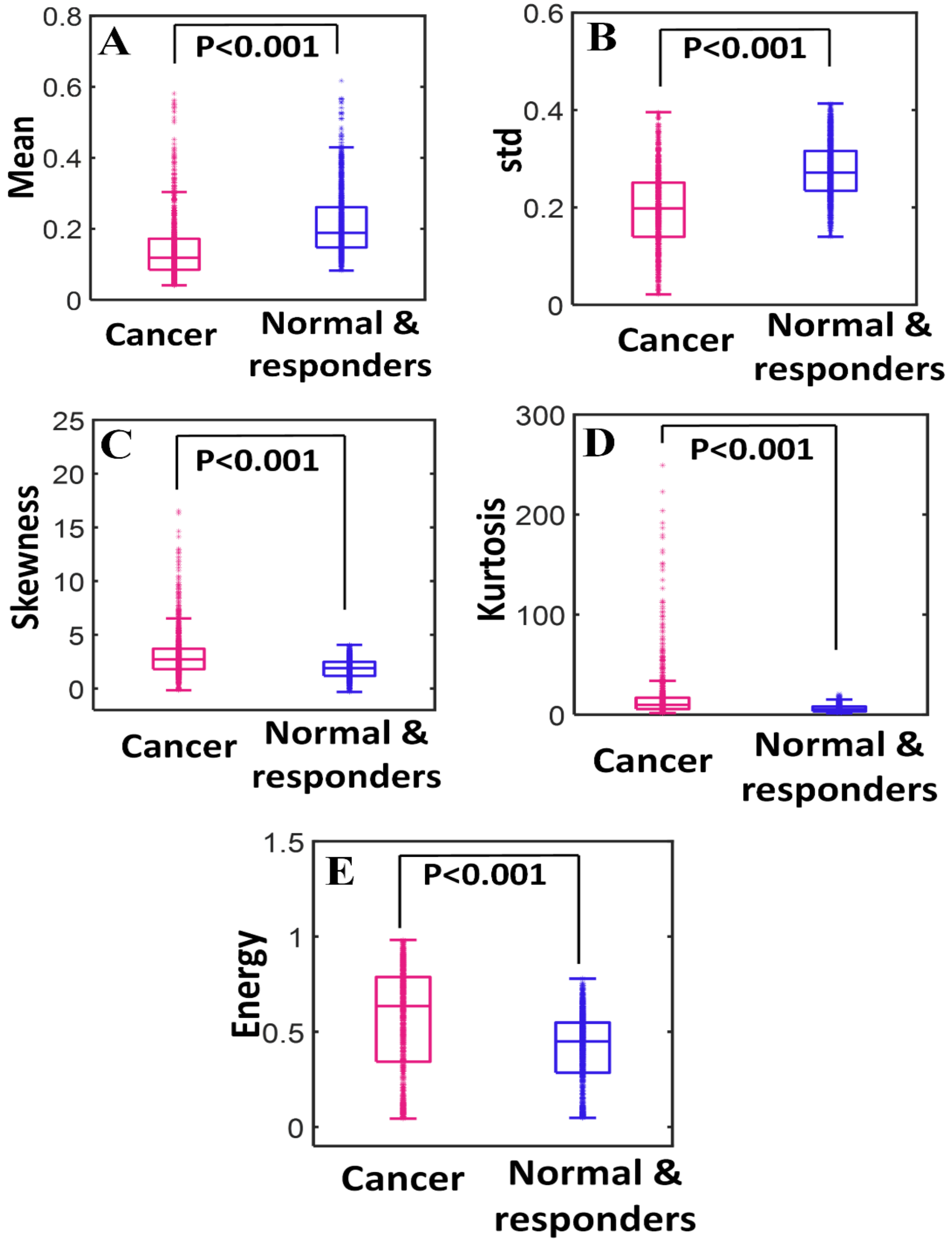

Figure S1. Boxplots of histogram features (Y axes) of PAM images. Each plotted point represents the histogram feature in one ROI. The p-value for each feature is shown on the plot.
